# Three-dimensional printing for assessment of paravalvular leak in transcatheter aortic valve implantation

**DOI:** 10.1186/s13019-020-01255-3

**Published:** 2020-08-05

**Authors:** Casey Thorburn, Omar Abdel-Razek, Susan Fagan, Neil Pearce, Michael Furey, Scott Harris, Michael Bartellas, Corey Adams

**Affiliations:** 1 Discipline of General Surgery, Memorial University of Newfoundland, St. John’s, Canada; 2 Discipline of Cardiology, University of Ottawa, Memorial University, Ottawa, Canada; 3 Department of Cardiology, Memorial University of Newfoundland, St. John’s, Canada; 4 Department of Radiology, Memorial University, St. John’s, Canada; 5 Memorial University of Newfoundland, St. John’s, Canada; 6 Department of Cardiac Surgery, Memorial University of Newfoundland, 300 Prince Philip Drive, St. John’s, NL A1B 3V6 Canada

**Keywords:** Aortic stenosis, Three-dimensional printing, Transcatheter aortic valve implantation

## Abstract

**Background:**

Three-dimensional (3D) models have the unique ability to replicate individualized cardiac anatomy and may therefore provide clinical benefit. Transcatheter aortic valve implantation (TAVI) currently relies on preoperative imaging for accurate valve sizing, type of valve used, and avoidance of complications. Three-dimensional (3D) modelling may provide benefit for optimal preoperative TAVI planning. The goal of this study is to assess the utility of 3D modelling in the prediction of paravalvular leak (PVL) post TAVI.

**Methods:**

Retrospective analysis of five patients who underwent TAVI at our center. Pre-operative cardiac gated CT images were utilized to create a 3D printed model with true size aortic root dimensions, including the coronary artery ostium location and left ventricular outflow tract. Deployment of the corresponding model and size TAVI valve into the created 3D model at a similar depth of implantation via fluoroscopy was performed for each patient. Degree of PVL was assessed using a closed system with water infusion under pressure over a duration of 5 s. Correlation was made between the volume obtained in the closed loop model during the pressurized period and the degree of PVL reported on the patients post TAVI placement on transthoracic echocardiogram.

**Results:**

One female, and four males (age in years ranged from 68 to 87) underwent successful TAVI (0% 30-day mortality). PVL on post procedure TTE ranged from none to trivial. Successful deployment of TAVI valves inside the 3D model occurred in all cases. The average volume of water collected on three trials over 5 s ranged between 19.1–24.1 ml A multivariate linear regression showed significant association between the degree of PVL reported on post-operative transthoracic echocardiogram and the amount of volume detected in the 3D model (difference: -3.9657, 95% CI: (− 4.6761,-3.2554), *p* < 0.001).

**Conclusions:**

Our experiments show that replicated 3D models have potential clinical utilization in predicting PVL in the TAVI population. Future research into the role of 3D modelling in the field of TAVI should continue to be explored.

## Background

Aortic Stenosis (AS) is the most common cause of valvular dysfunction in the Western world and has a significant impact on patient mortality and quality of life [1]. Transcatheter valve implantation (TAVI) is a mainstay of treatment for AS. Pre-operative imaging techniques are critical for successful TAVI, with incorrect decisions leading to potential valve embolization, paravalvular leak (PVL), and/or annular/aortic root rupture [2].

Currently, pre-operative cardiac imaging includes Computerized tomography (CT), Magnetic resonance imaging (MRI) and three-dimensional (3D) echocardiography, providing clinical insight into pre-procedural cardiac surgery planning. However, current imaging modalities do not always allow for perfectly accurate and intricate measurements of the patient’s anatomy and may not provide the exact contours of the aorta required for TAVI implantation [3]. Converting a patient’s 3D anatomy into a series of 2D images has the limitation of being less precise than true 3D recreation of the anatomy [4, 5]. The advent of 3D printing offers the ability to create precise models of a patient’s individual anatomy and may be even more effective in accurately assessing procedural complications and optimizing results [4, 5].

Three dimensional models of cardiac anatomy have demonstrated abilities in predicting outcomes of catheter-based procedures [6]. Ripley et al. demonstrated that 3D printed models provide a feasible, non-invasive technique to assist 3D visualization of patient-specific aortic root anatomy in a case series of 16 patients. The measurements of annulus minimum and maximum diameter made on printed 3D models were correlated to measurements of the annulus made on Cardiac CT [7]. S. Maragiannis et al. were able to fabricate a series of fully functional aortic stenosis models implantable within a flow loop, replicating the entire aortic valve complex using flexible material [8]. Schmauss et al. reported that Cardiac CT enabled the creation of 3D models of the aortic annulus and surrounding structures, allowing for potentially safer valve deployment in TAVI of Edwards SAPIEN 3 valve [9].

The primary goal of this project is to retrospectively analyze 3D printed models in comparison to the assessment of PVL in vivo after TAVI deployment in order to determine if an accurate prediction of PVL can be made. We describe our experience in creating patient-specific 3D printed anatomic models, developing a closed loop pressure system, as well as a measurement technique for measuring PVL in that model.

## Methods

### Demographics and data collection

We performed a retrospective study of data collected from patients who underwent TAVI procedure prior to 2018 in St. John’s, Newfoundland and Labrador (NL), Canada. The Eastern Health Regional Health Authority governs the Health Sciences Centre, a tertiary care referral center for Cardiology and Cardiac Surgery in the province of NL. All relevant patient demographic echocardiography findings were reviewed. Additional data was extracted from the Alberta Provincial Project for Outcome Assessment in Coronary Heart Disease (APPROACH) database in NL. Ethics approval was obtained from the Health Research Ethics authority.

### Inclusion criteria

Five randomly selected patients who underwent successful TAVI deployment among the first 40 cases performed in NL were used for the study. The randomized patient selection was accomplished by assigning each of these cases a number using statistical software. The pre-operative TAVI chest CT scans used in the planning for deployment for clinical TAVI was used to construct the 3D model. The isolated aortic anatomy from the chest CT was reformatted into STL format (Standard Tessellation Language) and refined from a mesh form for printing.

### Statistical analysis

Analyses were performed using Statistical Analysis Software (SAS) to complete a multivariate linear regression.

### Model development

**Fig. 1:**
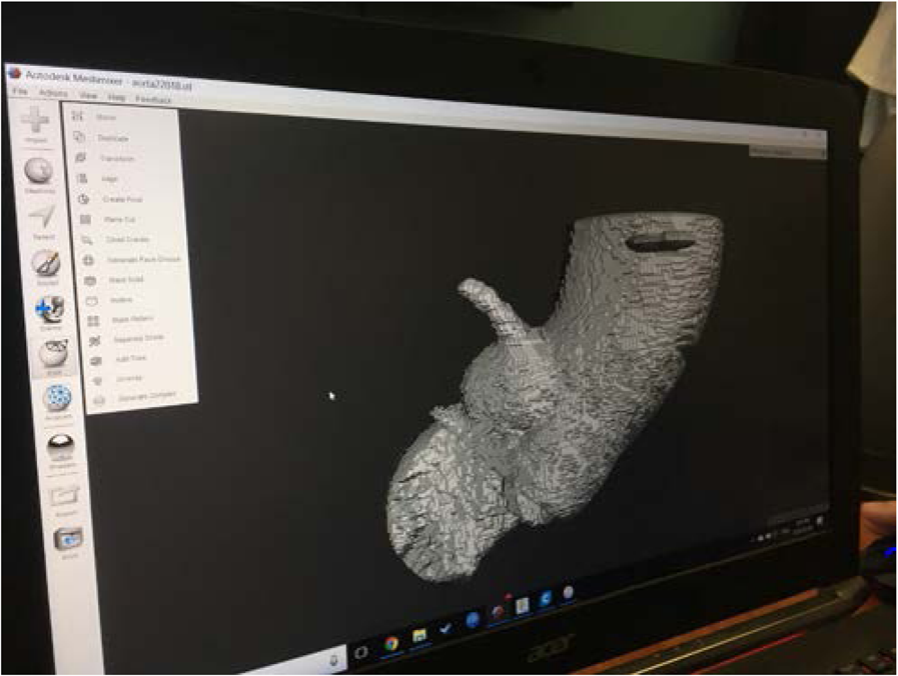
3D model STL format for printing

The pre-TAVI cardiac gated CT were de-identified and objects of interest were selected and separated within 3D data sets by segmentation in DICOM standard format. Aortic anatomy was isolated using Terra Recon Imaging and transferred to Standard Tessellation Language (STL) format for 3D cardiac printing. An example of this scan in STL format is seen in Fig. 1 below. Prints were developed using NinjaFlex thermoplastic polyurethane (TPU) material (Fenner Inc., Manheim, USA). Printing took approximately 4 h for each model. It was also necessary to print attachments in order to connect the aortic root proximally and distally to create a closed pressure system for testing of PVL once the respective aortic valve was implanted in each 3D model.

**Fig. 2:**
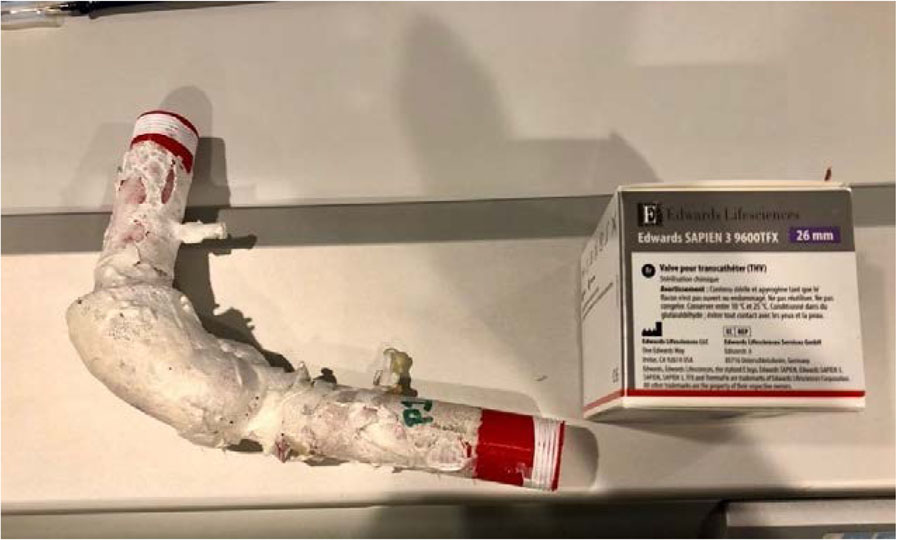
3D printed aortic model of patient 1 and corresponding valve

**Fig. 3:**
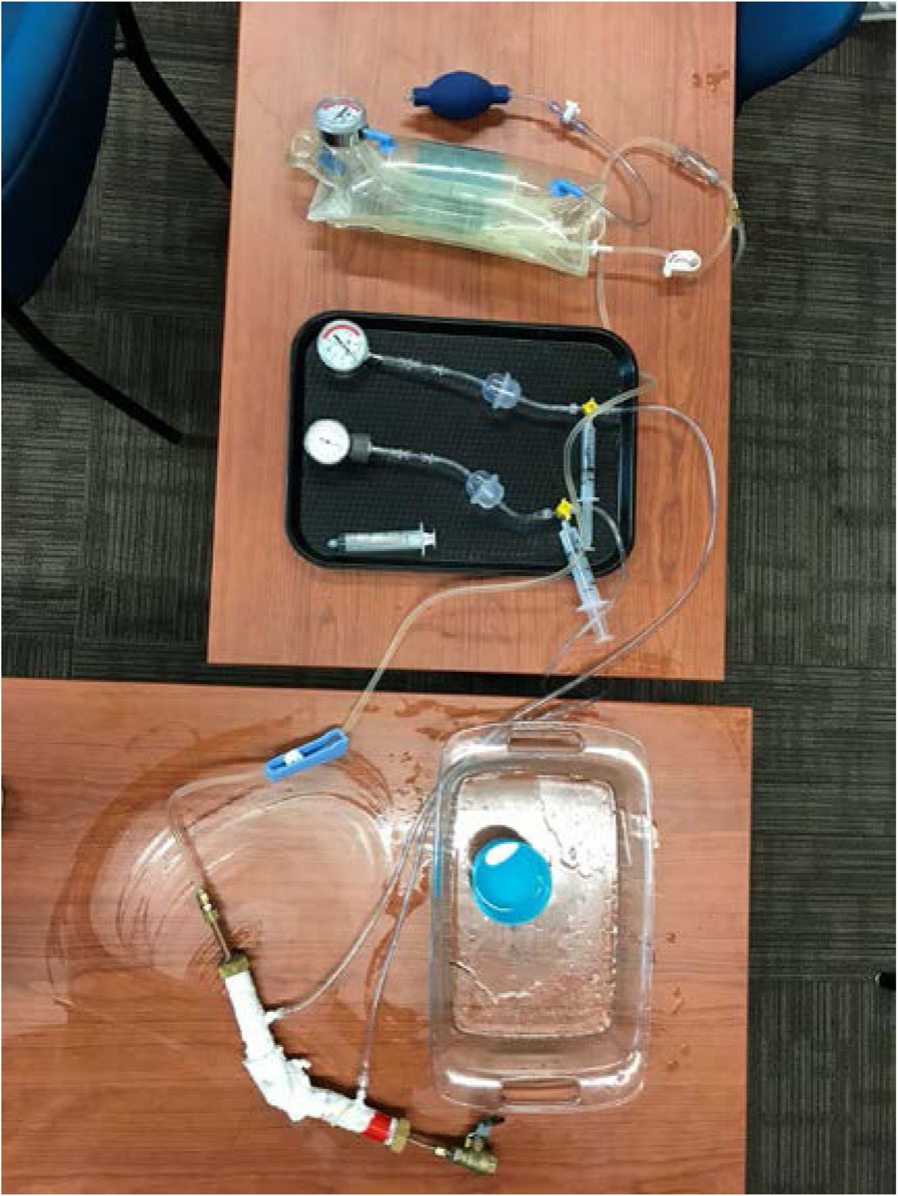
Closed pressure system for measuring paravalvular leak

**Fig. 4:**
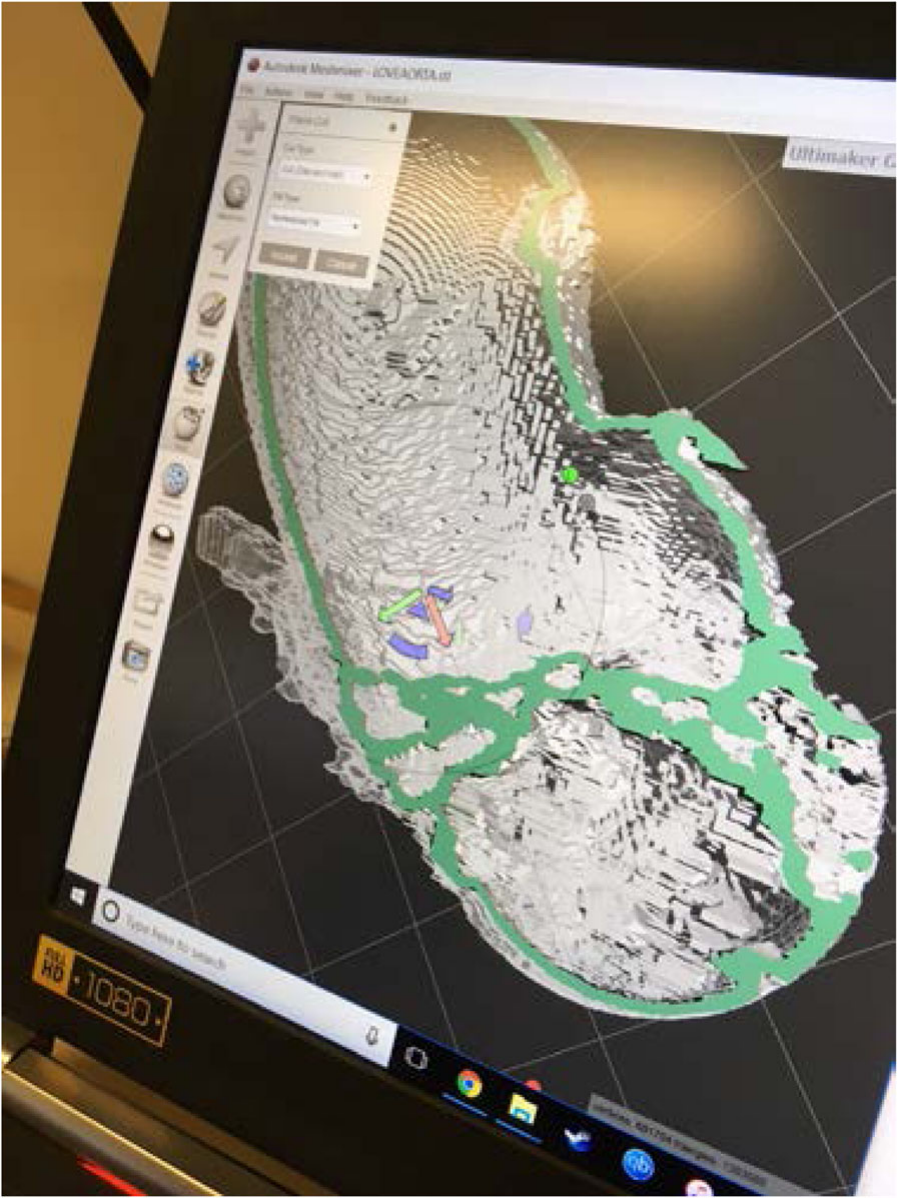
Smoothing of STL format aortic root prior to printing

The 3D cardiac printing was executed using a combination of three 3D printers: M3D micro(r), Ultimaker 2 + (r) and Lulzbot Taz 6(r). The final models were made of Ninjatek SemiFlex(r) or Ninjaflex (r). The 3D printed models comprised the aortic root, coronary ositum, and left ventricular outflow tract as see in Fig. 2. The current commercially approved sizes for the Edwards SAPIEN 3 TAVI valves are 23, 26, and 29. The appropriately sized models were implanted in each aortic 3D model respectively as in Fig. 3. STL format can leave gaps and small overlaps of the surface which were rectified by a 3D printing engineer (Fig. 4).

### Valve implantation

All implantations occurred under direct fluoroscopy and were performed with an interventional cardiologist and cardiac surgeon. The assumed aortic annulus on the 3D aortic root model was marked with a radiopaque marker. The depth of implant assessed on the post fluoroscopy super aortic root during patient procedure deployment was recreated when the valve was implanted within the root model.

### Model testing

Based on experimental data we determined the pressure across the TAVI valve which resulted in closure of the leaflets was a differential of 60 mmHg. Assuming that the valve was adequately closed at this pressure gradient any residual water obtained at the distal end would represent a paravalvular leak. Once the pressure gradient was sufficient to allow the valve to close, the system was opened distal to the valve and the amount of fluid was recorded over a time frame of 5 s. For each 3D printed model, three trials were performed and the amount of PVL in mililiters (ml) was recorded over 5 s. An average of the three trials was determined and the amount of leak was divided by five to give an average amount of leak in 1 s for each model. These amounts were compared to the amount of PVL recorded in post-operative echocardiogram for each patient by assigning the qualitative value of leak amount as a number from 0 to 4 to grade: 0 = none, 1 = trivial,2 = mild, 3 = moderate, and 4 = severe respectively. The presence or absence of PVL on the post-procedure TTE was confirmed by an experienced cardiologist with training in echocardiography, blinded to this study. All patients in the study received balloon-expandable Edwards Sapien valves (Sapien 3; Edwards LifeSciences, Irvine, CA). Prosthesis size in vivo was selected based on the annulus diameter, as measured by cardiac CT according to published recommendations, and all valves were maximally expanded.

### Data comparison to post-TAVI outcomes

A multivariate linear regression was performed which determined if the amount of paravalvular leak PVL found on post-operative echocardiogram could be effectively predicted by our estimates of paravalvular leak using 3D aortic models. The amount of leak described on post-operative echocardiogram was assigned a numerical amount as described above. The numerical values assigned to the qualitative description on post-operative echocardiogram were compared to the average amount of fluid per second recorded by our trials respectively for each patient.

## Results

The five patients selected included one female and four males. The age ranged from 68 to 87 years of age with an average age of 75. The patients had on average 3 comorbidities prior to aortic stenosis. The amount of leak reported on post-TAVI transthoracic echo was reported as ranging from none to trivial. The amount of leak from the 3D model was recorded as water collected over a 5 s interval then divided into the amount of paravalvular leak per second on average. The average amount of PVL of our models ranged from 19.1–24.1 with an average being 21.5 ml/second. Using a linear regression model controlled for the sex of the patient, the average volume recorded on the trials on the 3D model implantation was compared to the amount of leak assessed on the patient’s postoperative transthoracic echocardiogram. These results showed that the average amount of paravalvular leak of our 3D aortic models was significantly associated with the amount of leak on postoperative echocardiogram (*p* < 0.001).

## Discussion

The primary finding of this pilot study suggests that pre-operative 3D printing may have a potential utilization for assessing PVL. Our results demonstrated a significant correlation between early PVL reported on TTE to the amount of water in ml collected in our closed system within a 3D printed model. These results have promising opportunities for the usage of individually created 3D printed aortic root models to assist in improving clinical outcomes in TAVI.

The results of our 3D modelling study are similar to that of Ripley in 2016 which examined 16 patients retrospectively with a TAVI valve placed into the 3D printed aortic valve complex at the level of the annulus. Their study simulated PVL assessment using a light that was focused on the left ventricular side of the printed valve. Unimpeded light was able to pass through gaps between the aortic annulus and valve and PVL was detected by a digital camera positioned on the aortic side of the valve. In this study, paravalvular leak was correctly predicted in 6 of 9 patients and ruled out in 5 of the 7 patients (*P* = 0.13 by Chi Square analysis). The unique feature of our study is the utilization of a liquid medium in order to simulate and predict paravalvular leak. We feel a liquid medium gives a better representation of the true physiology and could be more applicable to PVL predictions in vivo. Our approach also utilized a closed system under pressure which we feel is also more representative of the true function of the valve.

Utilizing an individualized 3D model for TAVI cases has many potential benefits. Pre-procedure implantation simulation into an accurate 3D root could be invaluable in selecting valve size or type of valve (self-expanding vs balloon expanding) and assessing the impact of varying depth of implantation. Three-dimensional model would allow trainees to develop and improve implantation techniques in real time prior to the actual procedure. It could be used pre-operatively for simulation of varying valve implantation platforms and sizes. In addition, an accurate model could allow for improved education and trainee experience. The specific technical challenges of a TAVI implantation could be rehearsed with the clinical team in order to best prepare for such technical challenges.

There were several limitations to our study. Firstly, the same sample size is extremely small, however, the goal of this study was to generate a hypothesis and establish a proof of concept. Secondly, limitations in the creation of the closed system method for testing include using normal saline as our fluid which would have a different viscosity from blood and may have impacted our values for paravalvular leak. Thirdly, the material of the aortic roots themselves were not identical to the characteristic of a human aorta, nor was the elasticity or thickness compared to in vivo tissues. The material was slightly porous, and we had to use sealant during our experiment to maintain the integrity of the pressurized system. With advances in technology in the 3D printing field we anticipate the structural and anatomical fidelity of these models to greatly improve over the next several years. With regards to positioning of the TAVI valve, we attempted to deploy into the model at the same ventricular/aortic implant depth as we did during the patient’s actual procedure. Utilizing the fluoroscopy images during actual deployment we estimated the degree of implantation height around the “annulus” of the patient and attempted to recreate this during deployment into the 3D model. Differences in deployment height would be a limitation for PVL assessment, however we found doing this on the 3D models was simple and replication was very similar to the actual deployment depth. There was also no patient selected at random by our study with greater than a trivial amount of post-TAVI PVL. Having a greater range of PVL in future research would be beneficial to truly determine the accuracy of 3D modelling in predicting PVL. In addition, our model was static in that it was unable to replicate the cardiac expansion and pressure changes seen in diastole. However, we were able to maintain a minimum pressure gradient of 60mmhg above and below the valve which may be adequate as baseline testing with predictive value. The closed system measurement technique in this study could be improved with advances in technology such as an alternate to the porous material of the aortic root print itself, leak-proof attachments, improved pressure measurement accuracy, and utilization of different liquid medium with similar viscosity to blood.

Future studies aimed at deploying valves of different sizes, in different positions and orientations within identical versions of the same root may allow for pre-operative, patient-specific planning leading to the optimization of patient outcomes after TAVI.

## Conclusions

The results of this small study appear to show that 3D printing and modelling may have a benefit in procedural planning for TAVI and future studies should proceed.

## Data Availability

The dataset analyzed during this study are available in the APPROACH (Alberta Provincial Project for Outcome Assessment in Coronary Heart Disease) https://www.approach.org/about_pages/partners.html

## Abbreviations

PVL: Paravalvular leak
TAVI: Transcatheter aortic valve Implantation
CT: Computed tomography
US: Ultrasound
MRI: Magnetic resonance imaging
3D: Three-dimensional
